# Integrating System Dynamics Into Action Research: Drivers and Challenges in a Synergetic Complementarity: Comment on "Insights Gained From a Re-analysis of Five Improvement Cases in Healthcare Integrating System Dynamics Into Action Research"

**DOI:** 10.34172/ijhpm.2023.7585

**Published:** 2023-10-31

**Authors:** Mohammadreza Zolfagharian

**Affiliations:** Decision Sciences and Complex Systems Group, Faculty of Islamic Studies and Management, Imam Sadiq University, Tehran, Iran.

**Keywords:** Multimethodology, Mixed Methods, System Dynamics, Action Research, Modelling, Simulation

## Abstract

This commentary discusses the paper by Holmström et al that explored how system dynamics (SD) may contribute effectively to an action research (AR) process to improve five health case studies. Accordingly, we reviewed some of the methodological aspects of the proposed integration of SD into AR using ongoing debates on multi-methodology and mixed methods research. In a systemic evaluation of the proposed design, we concentrated on some of the common distinct features of SD and AR, and the challenges as well as the expected outcomes of this integration. Finally, we tried to position the suggested framework within the multi-methodology efforts and to pave the way for developing it in future research and practice.

## Introduction

 The paper by Holmström et al^1^ benefits a methodological enhancement in response to the challenges in implementing new policies in healthcare. In this research design, one central method (ie, action research [AR]) was used and enriched by selectively importing another method (ie, system dynamics [SD]). Accordingly, the authors integrated SD into AR in five case studies related to the health sector. In this regard, frameworks for consultancy assignments/client and socio-analytical questions were used to identify the project stages and bridge the two approaches. Finally, the authors identified and described general work principles and patterns whereby the synthesis of AR and SD can support the identification and implementation of workable solutions to the concerned challenges. As such, this complementarity was expected to exploit the benefits of both approaches holistically.

 In this commentary, we add to the perspective of Holmström et al and reflect more deeply upon the methodological approach of the paper. In particular, we aim to extend the methodological aspects of this paper using the ongoing mixed research and multi-methodology debates and inform the readers of the challenges raised in adopting such integrations.

## Drivers in Combining System Dynamics and Action Research

 Increasing critical awareness about the challenges and strengths of various approaches in dealing with different (dimensions and phases of) complex problematic situations has made the use of diverse methods and models more essential.^2,3^ Systemic evaluation of mixed research designs involves an investigation of the combined methods, the quality and the peculiarities of the integration, and the assessment of the achieved results (ie, emergent collective insights).^4^

 With regard to the latter, Holmström et al demonstrated that the proposed mixed-method approach derives, among others, the benefits of applying either approach in isolation. The authors reported more coherent, holistic, and robust outcomes with a higher likelihood of sustained actualization achieved through a deep engagement among the participants while gaining ownership and commitment to decisions (see also Box 1). However, critically evaluating and promoting the quality and impact^4,5^ of this integration might be considered as a promising direction for future applications. Interested readers can apply the related debates and frameworks in mixed methods research and multi-methodology interventions.^4-6^

**Box 1. The Added Value of Integrating System Dynamics Into Action Research^1,7^**

- More coherent, holistic, and robust outcomes
- Higher likelihood of sustained actualization
- More (objective) reflexivity about the concerned problem
- Feedback-oriented explanation of the problems
- Testing the impacts of improvement actions/scenarios *in silico*

To investigate the combined methods (ie, SD and AR), I want to emphasize on some striking similarities between these approaches that make their integration promising. Hence, the advantages of combining SD and AR can be considered mutual, whether SD integrates into AR as Holmström et al did or some of SD’s inputs can be taken from AR’s results. These common features are expected to be reinforced and synergetic, and provide emerging properties (cf. the outcomes) in case of their integration with each other.

 First, both approaches, with multiple paradigmatic underpinnings, can individually serve as a fertile ground for combining various quantitative, qualitative, and mixed methods.^8-10^

 Furthermore, systemic thinking is acknowledged as a grounding for AR that may broaden action and deepen research. That is, AR conducted with a systemic perspective in mind or built upon a formal systemic model, such as SD model, promises to construct meaning and action that resonates strongly with people’s experiences within a systemic world. As such, the cooperation of the stakeholders in the implementation phases would be more involved.^7^

 Finally, SD approach is almost conducive to eliciting, capturing, and changing stakeholders’ mental models through the modelling process.^11^ Similarly, AR also aims to transform the social reality through intervening in a problematic situation and changing the mindset of stakeholders.^8^

 In continue, I will point out to the quality and the peculiarities of the integration, by addressing the related challenges.

## Challenges of Integrating System Dynamics Into Action Research

 The proposed mixed research design by Holmström et al held off the most challenges of the mixed research design that might affect the feasibility and efficiency of multi-methodology interventions.^2^ Most importantly, philosophical challenges of integrating SD and AR can be mounted by adopting critical and transformative or pragmatist paradigms as the compatible philosophical underpinning of both approaches.^7,10-12^

 The similarities between SD and AR as well as work principles suggested in Holmström et al likely moderate the challenge on theoretical fitness of these approaches in terms of the coherence of integration across SD and AR as well as providing an added value for improving the concerned problematic situation and learning about it.

 However, practical challenges still exist in applying the proposed work principles in the health sector. Hence, Holmström et al^1^ demonstrated that “in none of the cases did the participants have to learn the basics of model building or SD terminology. However, they understood sufficiently [the basics of SD modelling] to see the simulation results as credible and useful.” Yet, the facilitator should express willingness and equip with SD and AR skills to engage efficiently with stakeholders over long periods.

 Acting in the framework of paradigms and methodologies requires knowing the (underpinnings of) research process along with bodily involvement, experience, and practice. As such, the cognition process preferences of the concerned researchers and stakeholders play a significant role in working across SD and AR and adopting the proposed design. This psychological challenge is quite significant in this design as AR and SD are both value-oriented but originate from different research traditions with distinct assumptions. As such, there might be cognitive barriers that lead to the resistance of researchers and practitioners to adopt the integration of SD into AR.

 Add to these challenges additional time and cost required in mixed research due to the need, among others, to collect and analyze two different types of data. This workload delays delivering an urgent solution to a complex problematic situation. You should also note the time-consuming nature of SD projects, as it can take weeks or even months for an expert modeler to create a robust and well-calibrated simulator.^11^ However, as Holmström et al^1^ claimed, virtual testing of the solutions via SD modelling process increases the overall time efficiency of this approach, and can outweigh a stand-alone AR approach.

 Furthermore, adopting this integration while engaging with stakeholders does not necessarily guarantee successful contributions to practical knowledge. On the one hand, not all interventions deliver helpful knowledge and solutions because the intervention might be poorly designed and conducted. On the other hand, there might be epistemological challenge raised by the participatory approaches that rely only on the co-production of knowledge between the participants.^10,13^ To moderate this challenge, the projects should always incorporate an understanding of the relevant extant literature/theories, as a profound foundation, with the lay knowledge of the participants in a coherent design. In this regard, using Brailsford’s three levels of implementation of simulations by Holmström et al^1^ seems to be instrumental. Of course, academic researchers, who aim to plan an AR design, should also avoid the trap of developing abstract theories with a cursory engagement with the practitioners without deeply involving them in the knowledge production process.

 Finally, the interactive nature of mixed methods research implies a reiterative cycle within the workflow.^6^ The authors indicated elsewhere this cyclical process; however, what we see in Figure 2, as the chronological workflows by case, is a sequential process that treats all cases, somehow, with the same beginning and end but includes different sequences of intermediate steps. Hence, both AR and SD involve dynamic, emergent, and continuous feedback processes that develop as those engaged, individually or collectively, deepen their understanding of the issues to be addressed.^8,11^ Accordingly, it is suggested to consider the whole process (rather than just individual steps) as an iterative and reciprocal cycle that guides the researchers and practitioners to engage in a reflective process.

 Overall, considering the above challenges and drivers, the choice to adopt various degrees of a mix between the SD and AR (as illustrated in Figure 1) largely depends on the initial research question, which in turn is likely to be influenced by the background, skills, and expertise of the facilitator/researcher in either/both approaches, interests of the stakeholders, and available time, data and other resources.^9^ Accordingly, problem owners might enjoy the consistency and the efficiency of a stand-alone approach due to the incompatibility of either one to the concerned situation. For example, if there is difficulty in the identification of the causal loop diagrams for the concerned problem, a pure AR or AR in combination with other approaches will be more rewarding.

## Challenges of the Participatory Approaches in Coercive Situations

 AR is an evident candidate approach when the objective is to explore theory related to practice and improve the concerned problem with the cooperation of related participants. However, it is necessary to consider the type of involved participants before adopting the proposed AR enhanced by SD.^14^ Holmström et al^1^ claimed that the groups in all case studies, except case 1, include Coalesced Authority, Power, and Influence required for decision-making and implementation of a solution. This group composition seems to be aligned with the participants in a pluralistic (rather than in a unitary or coercive) relationship. As such, the proposed mixed research design can converge the divergences in the opinions of the participants about the problems as well as their resolutions.

 Hence, those in a pluralist relationship differ in that, although their basic interests are compatible, they do not share the same values and beliefs. Space needs to be made available within which debate, disagreement, and even conflict, can take place. If this is done, and all feel they have been involved in decision‐making, then accommodations and compromises are accessible, at least temporarily. As such, the benefits of the mixed research will be somehow accessible.

 However, participants in coercive relationships with few shared interests would hold irreconcilable values and beliefs. In these situations, compromise is impossible, so no agreed objectives and subsequently no convergence can be obtained to direct action. Decisions are taken based on who has the most power, which is common in cases of tension between hierarchical power and the power of the professions rather than in cases of decentralized or democratic situations. In these problematic situations, divergence or creative phase might also be limited as the minorities with less power do not even feel safe to express their ideas in the meetings (absolutely if invited to). As such, alternative approaches should be adopted in order to comply with the peculiarities of these types of participants in the problematic situation.

## Towards a New Multi-methodology Framework

 In the multi-methodology debates, different frameworks have been suggested to guide method(s) selection in problematic situations. Two key frameworks can be identified in this regard^5,15^: (1) deductive or theory-led frameworks that derive from specific hypotheses or existing theories; and (2) inductive or practice-led frameworks that are generally built on the synthesis of method(s) selections in practices. The first type of framework was dominant in early debates on multi-methodology theory and practice. Yet, after the accumulation of interventions over the years, most recent frameworks are designed on the basis of synthesizing available evidence on combining methods.^15^

 The paper by Holmström et al^1^ described a method selection in practice deriving, particularly on reflexivity, group development, feedback-oriented explanation of the problems, and testing improvement actions *in silico*. Later on, this experience can be synthesized and complemented with other related methodological and practical evidences, likely leading to the construction of an inductive or practice-led framework. We hope considering the above issues, among others, helps pave the way towards improving health problems using the proposed research design as well as perhaps developing a more workable and effective multi-methodology framework.

## Ethical issues

 Not applicable.

## Competing interests

 Author declares that he has no competing interests.
